# Supplementary material for: DeepBiomarker2: Prediction of Alcohol and Substance Use Disorder Risk in Post-Traumatic Stress Disorder Patients Using Electronic Medical Records and Multiple Social Determinants of Health
Source: J Pers Med. 2024 Jan 14;14(1):94. doi: 10.3390/jpm14010094 (PMC10817272; doi:10.3390/jpm14010094)
Supplement: Supplementary file 1 [file jpm-14-00094-s001.zip › jpm-2746835-supplementary.pdf]

Supplementary information:

Supplementary information about SDoH parameters:

Racial segregation: Racial segregation was calculated as the index of concentration at the extremes (ICE) which is the measure of "social spatial polarization" that promotes the comparisons between neighborhoods which exhibit high degree of racial segregation. The measure was calculated by taking the difference in the total number of people occupying an advantaged vs disadvantaged position in society with respect to race and dividing by the total number of people in that geographic area[1]. The values of racial segregation range from -1 to 1, where -1 indicates a uniformly segregated disadvantaged area (low income) and 1 indicating a uniformly segregated advantaged area (high income). The formula is the numerator was the difference in the number of White households reporting ≥$100,000 or more income in the prior year (i.e., White households in highest income quintile) and Black households reporting ≤$25,000 (i.e., Black households in the lowest income quintile). We calculated zip code level information for each of the index dates and mapped them[2].

Income segregation: Like racial segregation, we also calculated income segregation based on the ICE principle. The formula is the summation of the number of all households reporting ≥$100,000 or more income in the prior year (i.e., highest income quintile) and those reporting ≤$25,000 (i.e. lowest income quintile). The difference between these two quantities (numerator) was divided by the total number of households in that zip code (denominator) to calculate the Income segregation score[2].

Neighborhood socio-economic status(nSES): To calculate nSES, we used the Agency for Healthcare Research and Quality (AHRQ) SES index formula[3]. The index was a weighted combination of the percentage of households with the mean number of 1 person or more per room, the median value of owner-occupied dwelling, the percentage unemployed, percentage living below the poverty level, the median household income, the percentage 25 years or older with a bachelor's degree or higher, and the percentage 25 years or older with less than a 12th-grade education. It is scaled to the US population to lie between 0 and 100, with a higher number indicative of greater neighborhood deprivation.

Formula: nSES= 50 + (0·11*median household income score) + (-0·10*% below federal poverty line) + (-0·08* % unemployed) + (0·10*% college graduates) + (-0·11* % education below 12th grade) + (0·08*median property value score) + (-0·07*% crowded households). Possible SES Index values range from 0 to 100, with higher values for the composite score representing higher SES levels.

Normalized difference vegetative index: We wanted to see if exposure to neighborhood greenness plays an impact on ASUD risk among PTSD patients. It was assessed using the Normalized Difference Vegetation Index (NDVI), a satellite-derived measure that captures photosynthetic activity of green, leafy vegetation. Values range from –1 to 1, with values between 0 and 1 corresponding to increasing levels of green leafy vegetation. Values below 0 indicate clouds, snow, or water. The data is obtained from the Landsat 5 and 7 satellites, which captured NDVI at 30 m resolution using Google Earth Engine. We used the nature equity dataset which is freely accessible[4].

Aridity index: To account for broad geographic patterns in NDVI, which varies based on climate and aridity in the given area, we included the Global Aridity Index as a variable in our model. We used the publicly available dataset which represents the ratio between precipitation and vegetation water demand, where lower values represent dry conditions[5].

Urban index: Based on U.S. census bureau, zip code is classified as predominately rural and urban, based on three key factors: population density (people per square mile), distance from nearest city, and size of the nearest city. We calculated and categorized each zip code based on the population density where, urban zip codes were categorized as 3,000+ persons per square mile. We used the nature equity dataset which is freely accessible[4].

Person of color index: Each zip code was categorized as white majority or people of color majority based on percentage of individuals living. We used the nature equity dataset which is freely accessible[4].

The variables: gender, age and race were extracted from each patient's EMR data.

The rest of the variables: percentage of households with same sex marriages, separated partners, single parent, no vehicle, limited English household, widowed partner is a male, percentage of US Citizens in a zip code, and % of non-citizens in a zip code, were collected from the ACS website(https://www.census.gov/).

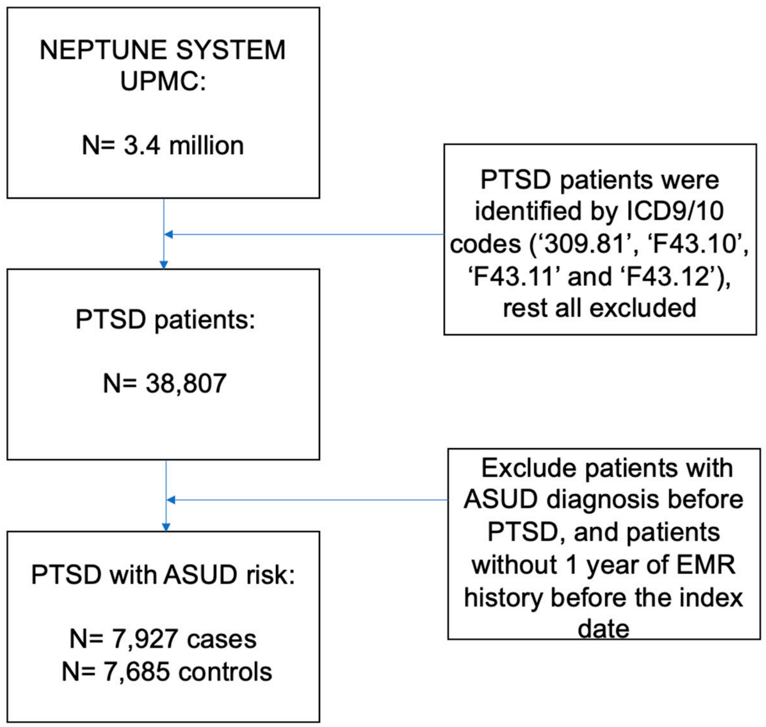

Figure S1: **CONSORT Diagram of our study**
PTSD: post-traumatic stress disorder, ASUD: alcohol and substance use disorder, ICD9/10: International classification of diseases 9/10, EMR: electronic medical records.

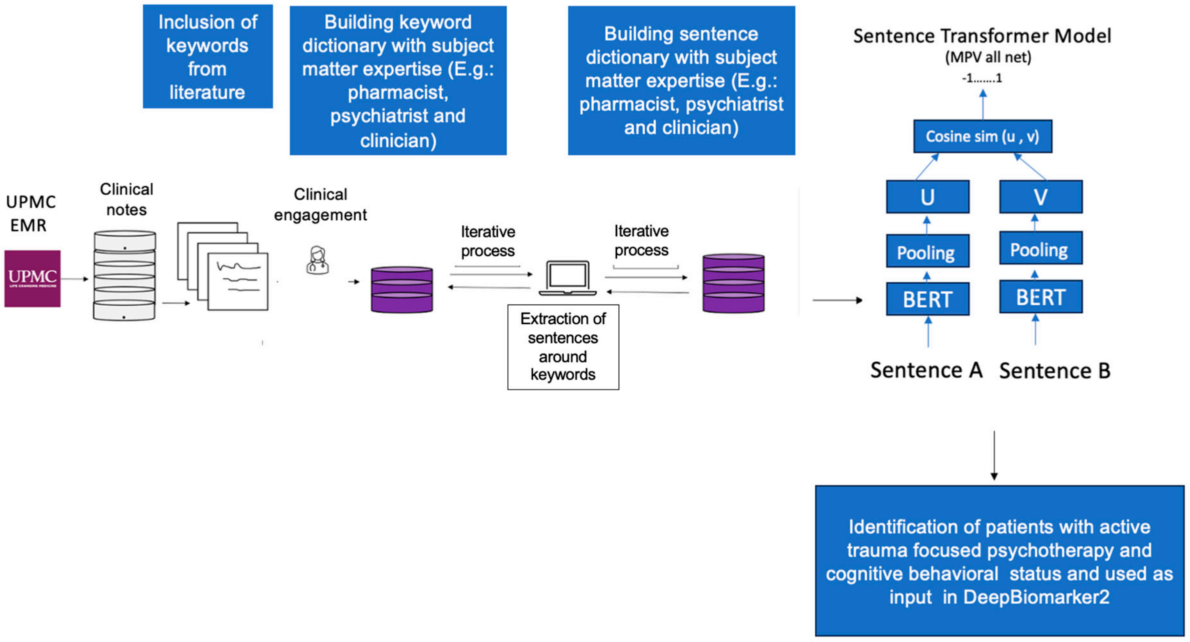

Figure S2: Iterative workflow of extraction of psychotherapy information from clinical notes

UPMC: University of Pittsburgh Medical Center, EMR: electronic medical records, BERT: Bidirectional encoder representations from transformers.

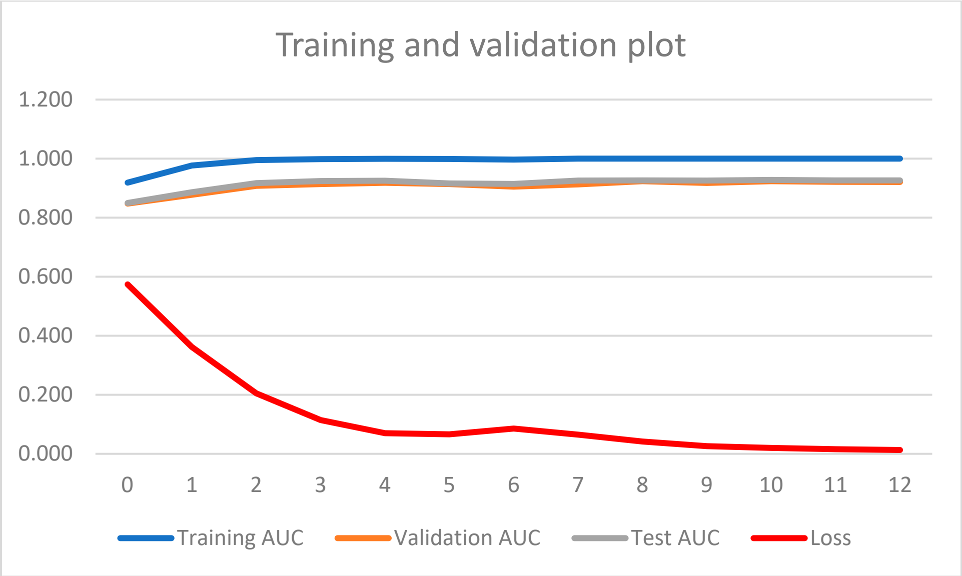

Figure S3: An example of training and validation plots for DeepBiomarker2. X axis is epoch. AUC: area under curve.

Supplementary information about medications impacting ASUD risk in PTSD patients

Our novel approach has proposed the following drugs may reduce the risk of ASUD among PTSD patients, an unexplored avenue until now. However, larger studies are needed to validate these findings for clinical application:

Diphenoxylate and Atropine: There is increasing research on the "gut-brain" axis which includes bidirectional signaling between the gut and the brain. Studies done showed greater PTSD and depressive symptoms severity was associated with worse constipation, diarrhea, gas/bloating, abdominal/belly pain[6, 7]. Diarrhea is also common phenomenon among intensive care patients. A systematic review identified diphenoxylate/atropine to be the most effective in treating these patients[8]. This drug is an opioid agonist primarily targeting mu receptors in the enteric nervous system, specifically the myenteric and submucosal plexus. It inhibits acetylcholine release, reducing gut motility and secretions and ultimately slowing down gastrointestinal transit. Studies have shown that care must be taken to avoid CNS effects including euphoria, nausea and dryness at higher doses[9]. However, atropine is given in combination with diphenoxylate to reduce its abuse potential.

Moxifloxacin: Moxifloxacin is a third-generation quinolone which has a broad antibacterial spectrum. It is used in the treatment of respiratory, intraabdominal, urinary tract, skin and soft tissue infections. It is less CNS adverse effects; however, two cases were reported where moxifloxacin induced hallucination[10] and acute psychosis[11] in patients with severe sepsis and community acquired pneumococcal pneumonia. Community acquired pneumonia is a common infection which is associated with significant mortality especially in elderly patients[12]. Initial antibacterial treatment is given to assure rapid clinical resolution and reduce high rates of hospitalization and mortality. moxifloxacin has demonstrated good clinical and bacteriological efficacy, reduced length of hospital stay and saved costs compared to standard therapy[13].

Valacyclovir: It is a highly effective and convenient treatment for acute herpes zoster. The I-valyl ester of acyclovir significantly enhances bioavailability, enabling simple dosing and improved outcomes[14]. In another study,

valacyclovir reduced herpes zoster-associated pain and postherpetic neuralgia, particularly in immunocompetent patients aged 50 years and above[15].

Xarelto/Rivaroxaban: It is a novel oral anticoagulant often referred to as a blood thinner, it is used for the treatment of venous thromboembolism, postoperative thromboprophylaxis following orthopedic surgeries, and preventing strokes in non-valvular atrial fibrillation. It has recently gained approval for use in secondary prevention for acute coronary syndrome and peripheral arterial disease[16]. While concerns exist about potential intracranial bleeding risks associated with rivaroxaban, a limited scale study found no significant differences in outcomes for patients with traumatic head injuries who received the treatment vs no treatment[17].

Sodium sulfacetamide sulfur: Dermatologic symptoms can be linked to PTSD in various ways: (i) as part of core PTSD symptoms, such as sensory flashbacks, night sweats, and dermatitis; (ii) due to the psychosomatic effects of abuse, neglect, and eating disorder; (iii) as a direct result of abuse or major life events; and (iv) through changes in the body's stress response, which can worsen skin conditions like psoriasis and urticaria. Stress-related inflammation and skin barrier issues may also play a role. Some PTSD patients have altered hormone levels and immune responses, further impacting their skin. These include hypothalamic-pituitary-adrenal axis hypo responsiveness and higher circulating T lymphocytes. PTSD should be considered when dealing with chronic or treatment-resistant stress-related skin issues and self-induced dermatoses[18]. Sodium sulfacetamide sulfur is an effective treatment for several inflammatory facial skin conditions and is frequently combined with sulfur for enhanced results. Adverse reactions to this medication are uncommon and usually involve only mild skin irritation. However, it should not be used by individuals with known sulfonamide allergies[19].

Table S1: The baseline comparison between case and control groups*

	Case (SD or %)	Control (SD or %)	p
n	7927	7685	
Age	35.16 (15.57)	36.1 (20.59)	1.33E-03
Gender Male	1884 (23.77)	2429 (31.61)	6.45E-28
309.81_posttraumatic stress disorder	4350 (54.88)	4004 (52.10)	5.12E-04
Emergence Department Visit	4616 (58.23)	3002 (39.06)	8.31E-127
300_Anxiety state, unspecified	3965 (50.02)	3293 (42.85)	2.74E-19
311_Depressive disorder, not elsewhere classified	4022 (50.74)	2709 (35.25)	5.52E-85
GLUCOSE	3229 (40.73)	1919 (24.97)	2.06E-97
724.2_Lumbago	2884 (36.38)	1554 (20.22)	6.10E-111
530.81_Esophageal reflux	2580 (32.55)	1787 (23.25)	2.89E-38
V15.82_Personal history of tobacco use	2498 (31.51)	1567 (20.39)	1.88E-56
V58.61_Long-term (current) use of anticoagulants	2291 (28.90)	1757 (22.86)	7.49E-18
R10.33_Periumbilical pain	2583 (32.58)	1407 (18.31)	6.58E-93
M25.50_Pain in unspecified joint	2413 (30.44)	1523 (19.82)	1.02E-52
M79.7_Fibromyalgia	2505 (31.60)	1345 (17.50)	8.53E-93
HEMATOCRIT(HCT)	2390 (30.15)	1429 (18.59)	2.79E-63
HGB	2402 (30.30)	1383 (18.00)	6.15E-72

V70.0_Routine general medical examination at a health care facility	1584 (19.98)	2185 (28.43)	5.95E-35
V72.83_Other specified pre-operative examination	2029 (25.60)	1632 (21.24)	1.29E-10
V76.2_Screening for malignant neoplasms of cervix	1947 (24.56)	1637 (21.30)	1.28E-06
RBC	2251 (28.40)	1321 (17.19)	2.31E-62
493.9_Asthma, unspecified type, unspecified	2172 (27.40)	1325 (17.24)	2.58E-52
RA ACETAMINOPHEN 500 MG CAPLET_DB00316	2276 (28.71)	1112 (14.47)	2.61E-103
272.4_Other and unspecified hyperlipidemia	1748 (22.05)	1633 (21.25)	2.24E-01
CHLORIDE(CL)	2085 (26.30)	1232 (16.03)	1.91E-55
338.29_Other chronic pain	2012 (25.38)	1249 (16.25)	1.04E-44
346.11_Migraine without aura, with intractable migraine, so stated, without mention of status migrainosus	1939 (24.46)	1247 (16.23)	2.65E-37
E849.0_Home accidents	1732 (21.85)	1447 (18.83)	2.79E-06
WBC	1937 (24.44)	1105 (14.38)	1.19E-56
278.01_Morbid obesity	1729 (21.81)	1288 (16.76)	1.33E-15
TOBRAMYCIN-DEXAMETH OPHTH SUSP_DB00684|DB01234	1662 (20.97)	1248 (16.24)	3.40E-14
ASPARTATE AMINOT.(AST)	1817 (22.92)	1028 (13.38)	8.13E-54
ABS NEUTROPHILS	1813 (22.87)	1031 (13.42)	7.36E-53
V11.8_Personal history of other mental disorders	1651 (20.83)	1170 (15.22)	9.32E-20
UREA NITROGEN	1766 (22.28)	990 (12.88)	1.79E-53
MCH	1703 (21.48)	922 (12.00)	1.54E-56
E006.9_Other activity involving other sports and athletics played individually	1318 (16.63)	1232 (16.03)	3.14E-01
ALANINE AMINOTRANS(ALT)	1740 (21.95)	809 (10.53)	4.79E-83
MCHC	1704 (21.50)	805 (10.47)	2.10E-78
296.32_Major depressive affective disorder, recurrent episode, moderate	1370 (17.28)	1076 (14.00)	1.71E-08
250_Diabetes mellitus without mention of complication, type II or unspecified type, not stated as uncontrolled	1512 (19.07)	916 (11.92)	6.05E-35
296.4_Bipolar I disorder, most recent episode (or current) manic, unspecified	1680 (21.19)	724 (9.42)	2.82E-92
Ventolin Hfa Aer Glax_DB01001	1444 (18.22)	951 (12.37)	4.27E-24
LEUKOCYTE ESTERASE	1592 (20.08)	783 (10.19)	2.24E-66
LYMPHOCYTES	1427 (18.00)	944 (12.28)	2.46E-23

V85.54_Body Mass Index, pediatric, greater than or equal to 95th percentile for age	1274 (16.07)	1082 (14.08)	5.08E-04
CALCIUM(CA)	1489 (18.78)	867 (11.28)	3.67E-39
RDW	1538 (19.40)	810 (10.54)	4.28E-54
BACTERIA	1633 (20.60)	713 (9.28)	3.39E-87
POTASSIUM(K)	1542 (19.45)	747 (9.72)	3.33E-66
V45.4_Arthrodesis status	1328 (16.75)	961 (12.50)	6.29E-14
OXYCODONE TAB 5MG_DB00497	1599 (20.17)	662 (8.61)	1.60E-93
715.9_Osteoarthrosis, unspecified whether generalized or localized, site unspecified	1332 (16.80)	922 (12.00)	1.32E-17
V73.81_Special screening examination for Human papillomavirus (HPV)	1293 (16.31)	947 (12.32)	1.18E-12
R53.83_Other fatigue	1284 (16.20)	943 (12.27)	2.30E-12
ABS BASOPHILS	1486 (18.75)	706 (9.19)	3.24E-66
786.5_Chest pain, unspecified	1353 (17.07)	797 (10.37)	6.44E-34
SODIUM(NA)	1446 (18.24)	656 (8.54)	1.41E-70
NEUTROPHILS	1290 (16.27)	781 (10.16)	2.22E-29
IBUPROFEN 600 MG TABLET_DB01050	1330 (16.78)	735 (9.56)	2.27E-40
285.9_Anemia, unspecified	1340 (16.90)	674 (8.77)	6.72E-52
MCV	1316 (16.60)	698 (9.08)	1.32E-44
BLOOD-URINE	1350 (17.03)	625 (8.13)	9.35E-63
Montelukast Sod 10 Mg Tabs_DB00471	1299 (16.39)	666 (8.67)	6.75E-48
TOTAL PROTEIN	1293 (16.31)	667 (8.68)	6.09E-47
793.89_Other (abnormal) findings on radiological examination of breast	1105 (13.94)	852 (11.09)	7.34E-08
ONDANSETRON ODT 4 MG TABLET_DB00904	1253 (15.81)	695 (9.04)	2.01E-37
HYDROCODONE-HOMATROPINE SYRUP_DB00956	1379 (17.40)	539 (7.01)	6.98E-87
CREATININE	1106 (13.95)	744 (9.68)	1.52E-16
599_Urinary tract infection, site not specified	1181 (14.90)	663 (8.63)	6.65E-34
MEDROXYPROGESTERONE 10 MG TAB_DB00603	871 (10.99)	972 (12.65)	1.31E-03
ALBUMIN	1186 (14.96)	636 (8.28)	1.11E-38
786.09_Other respiratory abnormalities	1135 (14.32)	686 (8.93)	9.36E-26
GABAPENTIN 100 MG CAPSULE_DB00996	1198 (15.11)	615 (8.00)	1.05E-43
ABS MONOCYTES	1186 (14.96)	613 (7.98)	1.64E-42
RA VITAMIN B-12 1,000 MCG TAB_DB00115	1012 (12.77)	777 (10.11)	1.90E-07

PROTEIN-URINE	1208 (15.24)	563 (7.33)	8.94E-55
PREDNISONE TAB 10MG_DB00635	1046 (13.20)	707 (9.20)	2.67E-15
CARBON DIOXIDE(CO2)	1129 (14.24)	603 (7.85)	4.48E-37
ANION GAP	1019 (12.85)	684 (8.90)	2.31E-15
V82.9_Screening for unspecified condition	1050 (13.25)	645 (8.39)	1.95E-22
SERTRALINE TAB 50MG_DB01104	811 (10.23)	866 (11.27)	3.63E-02
V15.88_History of fall	1040 (13.12)	625 (8.13)	5.97E-24
HIGH DENSITY LIPOPROTEIN(HDL)	1022 (12.89)	622 (8.09)	1.57E-22
ABS LYMPHOCYTES	1015 (12.80)	606 (7.89)	7.28E-24
CLONIDINE TAB 0.1MG_DB00575	1003 (12.65)	585 (7.61)	2.08E-25
564.89_Other functional disorders of intestine	952 (12.01)	625 (8.13)	9.25E-16
PLATELETS	977 (12.32)	578 (7.52)	1.24E-23
268.9_Unspecified vitamin D deficiency	768 (9.69)	777 (10.11)	3.77E-01
TRAZODONE 50 MG TABLET_DB00656	968 (12.21)	545 (7.09)	3.08E-27
GLUCOSE(BEDSIDE TEST)	998 (12.59)	511 (6.65)	3.58E-36
KETONES - URINE	987 (12.45)	509 (6.62)	3.91E-35
NEXIUM CAP 40MG_DB00736|DB14513	834 (10.52)	645 (8.39)	5.65E-06
HEPARIN SOD 20,000 UNIT/ML VL_DB01109	1042 (13.14)	429 (5.58)	8.10E-59
314.01_Attention deficit disorder with hyperactivity	702 (8.86)	755 (9.82)	3.75E-02
SUPREP BOWEL PREP KIT_DB14500	715 (9.02)	735 (9.56)	2.41E-01
HYDROXYZ HCL TAB 25MG_DB00557	912 (11.50)	522 (6.79)	2.14E-24
787.91_Diarrhea	915 (11.54)	504 (6.56)	2.43E-27
465.9_Acute upper respiratory infections of unspecified site	758 (9.56)	646 (8.41)	1.16E-02
780.99_Other general symptoms	944 (11.91)	443 (5.76)	1.80E-41
WHITE BLOOD CELLS-URINE	936 (11.81)	440 (5.73)	5.88E-41
EPITHELIAL CELLS	967 (12.20)	409 (5.32)	7.29E-52
V65.3_Dietary surveillance and counseling	518 (6.53)	849 (11.05)	1.99E-23

Only the top 100 most frequent features collected during the 1-year observation period are listed in this table. The p value is from t-test for continuous feature (age) and chi-square test for category features (others).

Table S2: Important features identified by perturbation-based contribution analysis for ASUD prediction:

Feature Name	Relative Contribution	Wilcoxon_p	FDR_Q

ED Visit_ED Visit	1·50	1·79E-65	1·79E-65
RA ACETAMINOPHEN 500 MG CAPLET_DB00316	1·60	3·67E-38	1·84E-38
HGB	1·46	2·63E-34	8·75E-35
HEMATOCRIT(HCT)	1·40	5·43E-29	1·36E-29
GLUCOSE	1·32	9·64E-28	1·93E-28
V70.0_Routine general medical examination at a health care facility	0·71	9·76E-25	1·63E-25
V65.3_Dietary surveillance and counseling	0·47	7·63E-24	1·09E-24
530.81_Esophageal reflux	1·25	1·40E-16	1·68E-17
493.9_Asthma, unspecified type, unspecified	1·34	1·51E-16	1·68E-17
RBC	1·28	4·99E-16	4·99E-17
WBC	1·32	6·02E-14	5·47E-15
CHLORIDE(CL)	1·29	8·99E-14	7·49E-15
MCHC	1·34	9·79E-13	7·53E-14
314.01_Attention deficit disorder with hyperactivity	0·50	3·00E-12	2·14E-13
MCH	1·31	4·18E-11	2·78E-12
ALBUMIN	1·35	5·77E-11	3·61E-12
RDW	1·30	7·80E-11	4·59E-12
HYDROCODONE-HOMATROPINE SYRUP_DB00956	1·42	1·38E-10	7·66E-12
OXYCODONE TAB 5MG_DB00497	1·44	3·49E-10	1·84E-11
TOTAL PROTEIN	1·41	1·31E-09	6·57E-11
646.84_Other specified complications of pregnancy, postpartum condition or complication	2·10	1·39E-09	6·60E-11
PROTEIN-URINE	1·41	2·20E-09	1·00E-10
V58.61_Long-term (current) use of anticoagulants	1·24	4·82E-09	2·10E-10
382.9_Unspecified otitis media	0·44	5·39E-09	2·25E-10
V15.82_Personal history of tobacco use	1·25	8·78E-09	3·51E-10
LEUKOCYTE ESTERASE	1·36	9·21E-09	3·54E-10
300_Anxiety state, unspecified	1·18	1·21E-08	4·49E-10

ABS NEUTROPHILS	1·33	1·95E-08	6·95E-10
DIPHENOXYLATE ATROPINE 2 5_DB00572|DB01081	0·30	4·07E-08	1·40E-09
579.2_Blind loop syndrome	0·24	6·52E-08	2·17E-09
780.6_Fever, unspecified	0·47	7·04E-08	2·27E-09
Sod Sul/Sulf 10-5 % Emu Exac_DB00634	0·45	2·52E-07	7·87E-09
GABAPENTIN 100 MG CAPSULE_DB00996	1·39	4·16E-07	1·26E-08
250_Diabetes mellitus without mention of complication, type II or unspecified type, not stated as uncontrolled	2·17	4·72E-07	1·39E-08
852.02_Subarachnoid hemorrhage following injury without mention of open intracranial wound, with brief [less than one hour] loss of consciousness	0·50	4·97E-07	1·42E-08
724.2_Lumbago	1·25	8·80E-07	2·45E-08
V11.8_Personal history of other mental disorders	1·28	1·15E-06	3·10E-08
844.9_Sprains and strains of unspecified site of knee and leg	0·41	2·09E-06	5·50E-08
311_Depressive disorder, not elsewhere classified	1·21	4·54E-06	1·16E-07
240.9_Goiter, unspecified	0·54	5·33E-06	1·33E-07
Z3A.38_38 weeks gestation of pregnancy	1·34	7·51E-06	1·83E-07
386.11_Benign paroxysmal positional vertigo	0·34	1·84E-05	4·38E-07
R10.33_Periumbilical pain	1·17	1·93E-05	4·48E-07
Flecainide 50 Mg Tab Anip_DB01195	0·08	2·24E-05	5·09E-07
738_Acquired deformity of nose	0·14	5·58E-05	1·24E-06
403.1_Hypertensive chronic kidney disease, benign, with chronic kidney disease stage I through stage IV, or unspecified	2·27	9·52E-05	2·07E-06
TRULANCE 3 MG TABLET_DB13170	0·19	0·0001	2·20E-06
ENALAPRIL 20MG TABLETS_DB00584	0·48	0·0001	2·84E-06
CARBON DIOXIDE(CO2)	1·28	0·0001	2·87E-06
UREA NITROGEN	1·20	0·0001	2·87E-06
780.99_Other general symptoms	0·67	0·0002	3·73E-06
HIGH SENSITIVITY CRP	1·84	0·0002	3·80E-06
646.93_Unspecified complication of pregnancy, antepartum condition or complication	1·36	0·0002	3·81E-06
615_Acute inflammatory diseases of uterus, except cervix	2·21	0·0002	3·81E-06

846_Sprain of lumbosacral (joint) (ligament)	2·88	0·0003	5·19E-06
MOXIFLOXACIN HCL 400 MG TABLET_DB00218	0·31	0·0003	5·46E-06
CALCIUM(CA)	1·27	0·0003	5·77E-06
LACTATE ISTAT	0·39	0·0004	6·13E-06
873.44_Open wound of jaw, without mention of complication	0·35	0·0004	7·52E-06
793.7_Nonspecific (abnormal) findings on radiological and other examination of musculoskeletal system	0·18	0·0005	7·94E-06
706.1_Other acne	0·66	0·0013	2·12E-05
560.9_Unspecified intestinal obstruction	0·36	0·0017	2·70E-05
648.14_Thyroid dysfunction of mother, postpartum condition or complication	0·17	0·0017	2·74E-05
Alprazolam 1 Mg Tab Dava_DB00404	1·63	0·0019	2·89E-05
E929.0_Late effects of motor vehicle accident	0·56	0·0022	3·42E-05
M79.7_Fibromyalgia	1·19	0·0024	3·58E-05
EPITHELIAL CELLS	1·26	0·0037	5·56E-05
577_Acute pancreatitis	0·67	0·0039	5·66E-05
MCV	1·18	0·0043	6·17E-05
211.3_Benign neoplasm of colon	0·59	0·0050	7·08E-05
NADOLOL TAB 20MG_DB01203	0·15	0·0052	7·37E-05
BACTERIA	1·18	0·0056	7·73E-05
296.4_Bipolar I disorder, most recent episode (or current) manic, unspecified	1·26	0·0074	0·0001
DICLOFENAC SOD EC 75 MG TAB_DB00586	3·29	0·0095	0·0001
ANA PATTERN	0·19	0·0106	0·0001
C75.1_Malignant neoplasm of pituitary gland	4·08	0·0111	0·0001
LYMPHOCYTES	1·20	0·0134	0·0002
251.2_Hypoglycemia, unspecified	2·33	0·0144	0·0002
346.11_Migraine without aura, with intractable migraine, so stated, without mention of status migrainosus	1·17	0·0146	0·0002
COLESTIPOL HCL 1 GM TABLET_DB00375	0·16	0·0150	0·0002
715.9_Osteoarthrosis, unspecified whether generalized or localized, site unspecified	1·26	0·0157	0·0002
BUSPIRONE HCL 5 MG TABLET_DB00490	2·37	0·0163	0·0002
V45.4_Arthrodesis status	1·18	0·0192	0·0002

787.2_Dysphagia, unspecified	0·47	0·0196	0·0002
UA CULTURE SCREEN	0·46	0·0224	0·0003
SYMBICORT 80-4.5 MCG INHALER_DB00983|DB01222	1·36	0·0228	0·0003
V85.54_Body Mass Index, pediatric, greater than or equal to 95th percentile for age	0·86	0·0261	0·0003
ASPARTATE AMINOT.(AST)	1·19	0·0285	0·0003
785.2_Undiagnosed cardiac murmurs	0·42	0·0294	0·0003
CLINDAMYCIN HCL 150 MG CAPSULE_DB01190	0·59	0·0295	0·0003
434.91_Cerebral artery occlusion, unspecified with cerebral infarction	0·24	0·0330	0·0004
BASE DEFICIT-ARTERIAL &&	1·76	0·0332	0·0004
XARELTO 20MG TABLETS_DB06228	0·39	0·0337	0·0004
BLOOD-URINE	1·21	0·0418	0·0004
V58.49_Other specified aftercare following surgery	1·92	0·0432	0·0005
959.3_Elbow, forearm, and wrist injury	0·44	0·0442	0·0005
IONIZED CALCIUM, ISTAT	0·44	0·0444	0·0005
VALACYCLOVIR HCL 1 GRAM TABLET_DB00577	0·53	0·0495	0·0005
V18.19_Family history of other endocrine and metabolic diseases	0·57	0·0509	0·0005
LDL CHOLESTEROL, DIRECT	0·12	0·0535	0·0005
614.9_Unspecified inflammatory disease of female pelvic organs and tissues	0·49	0·0678	0·0007
338.29_Other chronic pain	1·17	0·0704	0·0007
A/G RATIO	1·35	0·0707	0·0007
ALPHA 1	0·19	0·0730	0·0007
PENICILLN VK TAB 500MG_DB00417|DB14500	0·65	0·0735	0·0007
646.13_Edema or excessive weight gain in pregnancy, without mention of hypertension, antepartum condition or complication	3·12	0·0769	0·0007
GLIPIZIDE 5 MG TABLET_DB01067	2·34	0·0819	0·0008
IGE-TOTAL	14·80	0·0857	0·0008
618.4_Uterovaginal prolapse, unspecified	0·28	0·0938	0·0009
351_Bell's palsy	1·77	0·0948	0·0009
V72.42_Pregnancy examination or test, positive result	1·31	0·0994	0·0009
217_Benign neoplasm of breast	0·21	0·1142	0·0010
EPSTEIN-BARR VCA IGG AB	0·33	0·1206	0·0011
401.9_Unspecified essential hypertension	1·33	0·1211	0·0011
ASPIR-LOW EC 81 MG TABLET_DB00945	1·39	0·1257	0·0011
368.8_Other specified visual disturbances	1·64	0·1268	0·0011
NOROXYCODONE	2·84	0·1273	0·0011
GARLIC 1,000 MG CAPSULE_DB10532	2·19	0·1327	0·0011

HEMATOCRIT (POCT)	0·17	0·1336	0·0011
V15.3_Personal history of irradiation, presenting hazards to health	0·62	0·1381	0·0012
V46.8_Dependence on other enabling machines	0·52	0·1435	0·0012
V58.89_Other specified aftercare	0·38	0·1527	0·0013
FENOFIBRIC ACID DR 135 MG CAP_DB00122|DB13873	0·27	0·1604	0·0013
ZINC (ZN)	0·56	0·1682	0·0014
ELMIRON CAP 100MG_DB00686	0·51	0·1695	0·0014
VERY LOW DENSITY LIPOPROTEIN	0·55	0·1794	0·0014
URINE YEAST	0·36	0·1811	0·0014
M62.830_Muscle spasm of back	0·67	0·1847	0·0014
PH ISTAT VENOUS	0·52	0·1874	0·0014
695.89_Other specified erythematous conditions	0·16	0·1875	0·0014
METRONIDAZOL TAB 500MG_DB00916	1·27	0·1931	0·0015
GLUCOSE-URINE	1·27	0·1968	0·0015
PLATELETS	1·23	0·1976	0·0015
V22.1_Supervision of other normal pregnancy	1·25	0·2155	0·0016
COSENTYX PEN INJ 300DOSE_DB09029	2·59	0·2181	0·0016
842_Sprain of wrist, unspecified site	0·61	0·2268	0·0017
722.52_Degeneration of lumbar or lumbosacral intervertebral disc	1·48	0·2276	0·0017
NEOMYCIN-POLYMYXIN-HC EAR SUSP_DB00994|DB00781	0·44	0·2351	0·0017
227_Benign neoplasm of adrenal gland	2·84	0·2717	0·0020
313.89_Other emotional disturbances of childhood or adolescence	1·33	0·3173	0·0023
847_Sprain of neck	3·18	0·3411	0·0024
ACEBUTOLOL 200 MG CAPSULE_DB01193	0·40	0·3503	0·0025
848.3_Sprain of ribs	0·58	0·3622	0·0025
875.1_Open wound of chest (wall), complicated	0·44	0·3710	0·0026
657.03_Polyhydramnios, antepartum condition or complication	16·65	0·3732	0·0026
959.9_Unspecified site injury	0·63	0·3852	0·0026
LACTASE ENZYME 3000UNIT TABS_DB13761	0·12	0·3904	0·0027
ACETAZOLAMIDE 125 MG TABLET_DB00819	1·65	0·4083	0·0028
V25.11_Encounter for insertion of intrauterine contraceptive device	1·46	0·4123	0·0028
285.29_Anemia of other chronic disease	0·40	0·4591	0·0031
INTACT PTH	2·06	0·4647	0·0031
V01.89_Contact with or exposure to other communicable diseases	1·34	0·4802	0·0032
959.01_Head injury, unspecified	0·71	0·4866	0·0032
V54.89_Other orthopedic aftercare	0·30	0·4874	0·0032

576.1_Cholangitis	0·49	0·4977	0·0032
658.13_Premature rupture of membranes, antepartum condition or complication	0·38	0·5067	0·0032
PREZISTA TAB 600MG_DB00898|DB01264	0·39	0·5437	0·0035
CANNABINOID	0·28	0·5483	0·0035
E980.4_Poisoning by other specified drugs and medicinal substances, undetermined whether accidentally or purposely inflicted	0·50	0·5602	0·0035
B-TYPE NATRIURETIC PEPTIDE	1·54	0·5733	0·0036
LORAZEPAM 1 MG TABLET_DB00186	0·39	0·5858	0·0036
V76.2_Screening for malignant neoplasms of cervix	0·86	0·5997	0·0037
331.83_Mild cognitive impairment, so stated	2·61	0·6166	0·0038
192.2_Malignant neoplasm of spinal cord	0·57	0·6195	0·0038
591_Hydronephrosis	1·60	0·6215	0·0038
536.42_Mechanical complication of gastrostomy	0·59	0·6233	0·0038
651.03_Twin pregnancy, antepartum condition or complication	0·10	0·6303	0·0038
536.3_Gastroparesis	0·21	0·6433	0·0038
380.1_Infective otitis externa, unspecified	0·46	0·6513	0·0039
E929.0_Late effects of motor vehicle accident	2·59	0·6602	0·0039
AZATHIOPRINE 50 MG TABLET_DB00993	0·66	0·6657	0·0039
SYPHILIS SCREEN	0·05	0·6751	0·0039
BETAMETHASONE DP 0.05% OINT_DB00443|DB11130	0·81	0·6765	0·0039
E920.8_Accidents caused by other specified cutting and piercing instruments or objects	0·66	0·6779	0·0039
787.3_Flatulence, eructation, and gas pain	0·65	0·7093	0·0040
SODIUM(NA)	1·16	0·7093	0·0040
437.8_Other ill-defined cerebrovascular disease	2·26	0·7576	0·0043
CANDIDA PROBE	0·63	0·7768	0·0044
MAGNESIUM OXIDE 400 MG TABLET_DB01377	0·75	0·7895	0·0044
POTASSIUM(K)	1·15	0·8905	0·0049
E812.0_Other motor vehicle traffic accident involving collision with motor vehicle injuring driver of motor vehicle other than motorcycle	1·83	0·9212	0·0051
ANA SCREEN	2·56	0·9223	0·0051
RA P-COL RITE TABLET_DB11089	1·20	0·9582	0·0052

Relative contribution value > 1: Indicator of high risk and Relative contribution value < 1: Indicator of low risk, FDR_Q: false discovery rate adjusted Q value, p_wilcoxon: P values of Wilcoxon test. The diagnostic codes mentioned in the table above (Diagnosis code/DrugBank ID/lab-tests) used in our study are representatives of all 1639 diagnosis groups.

Table S3: Association of lab tests, diagnosis and medication use on PTSD and ASUD validated by literature search.

Lab test	Effect on PTSD	Effect on ASUD	Diagnosis	Medication use
**Heme:**				
HGB	High hemoglobin levels[20]	Low hemoglobin in alcoholics, opioids, cannabis and heroin users[21]	Long term (current) use of anticoagulants[22, 23]	
HCT	High hematocrit promoting inflammation[24]	Low hematocrit in alcoholics, cannabis, cocaine users and high hematocrit levels in opioid users[21]	Long term (current) use of anticoagulants[22, 23]	
RDW	Higher RDW levels amongst depressed patients[24]	High RDW in cannabis users and heroin users and low RDW in opioid users[21]	Long term (current) use of anticoagulants[22, 23]	
RBC	Elevated RBCs in depressed patients[24]	Low red blood cells in alcoholics, opioids and heroin users and high red blood cells in cannabis users[21]	Long term (current) use of anticoagulants[22, 23]	
MCH	Negative association between MCH and anxiety/depression[24]	Significant elevations in MCH and MCHC in severe alcoholics and cannabis users. But slight elevations in moderate alcoholics[21]	Long term (current) use of anticoagulants[22, 23]	

MCHC	High MCHC levels in trauma patients[24]	Significant elevations in MCH and MCHC in severe alcoholics, heroin and cannabis users. But slight elevations in moderate alcoholics[21]	Long term (current) use of anticoagulants[22, 23]	
**Kidney:**				
ALBUMIN	Low serum albumin levels in mood disorders (schizophrenia and MDD)[25]	Low serum albumin levels in drug addicts while higher serum albumin levels in alcoholics[26]	Acute encephalopathy for liver alcoholic diseases[27]	
UREA- NITROGEN	Significant relationship between depression and abnormal urea nitrogen[28]	Increased urea nitrogen in morphine users[29]		
CALCIUM	Abnormal calcium levels[30]	Multiple dyselectrolytemia in chronic alcohol abusers[31]		
CHLORIDE	Hyperchloremia[32]	Multiple dyselectrolytemia in chronic alcohol abusers[31]		

CARBON DIOXIDE	Abnormal carbon dioxide levels in head injury patients[33]	Increased partial pressure of arterial carbon dioxide levels in alcohol withdrawal patients[34]		
**Metabolic syndrome:**				
GLUCOSE	High blood glucose peaks and insulin levels in PTSD patients[35]	Glucose dysregulation amongst ASUD patients[36]	Diabetes mellitus[36]	
**Inflammatory:**				
WBC	Elevated WBCs in depressed patients[37]	Low WBC levels in alcoholics. But high elevations in cannabis, inhalants, tobacco, opioid users. No significant changes amongst cocaine users[21]	Major depressive disorder, lumbago, fibromyalgia, asthma, other chronic pain[38-42]	Pain medications[43]
ABS NEUTROPHILS	Elevated neutrophils in depressed patients[37]	Low neutrophil levels in alcoholics and cannabis users. But high elevations in inhalants and opioid users[21]	Major depressive disorder, lumbago, fibromyalgia, asthma, other chronic pain[38-42]	Pain medications[43]
**Urine:**				
LEUKOCYTE ESTERASE	Bacterial vaginosis increases blood urine content (BV)[44]	Poor quality of life by UTI patients increase risk of ASUDs[45]	Other general symptoms[46]	

Miscellaneous:				
TOTAL PROTEIN	High total protein content in trauma patients[47]	Low protein content due to poor nutrition habits amongst addicts[48]	Other general symptoms such as nausea, headache, low on energy[48]	

*Hemoglobin (HGB), hematocrit (HCT), red cell distribution width (RDW), red blood cells (RBC), white blood cells (WBC), absolute (ABS) neutrophils, mean corpuscular hemoglobin (MCH) and mean corpuscular hemoglobin concentration (MCHC).

REFERENCES:

1. Krieger N, W.P., Spasojevic J, Li W, Maduro G, Van Wye G, *Public Health Monitoring of Privilege and Deprivation With the Index of Concentration at the Extremes.* Am J Public Health, 2016.
2. Chambers, B.D.; Baer, R.J.; McLemore, M.R.; Jelliffe-Pawlowski, L.L. *Using Index of Concentration at the Extremes as Indicators of Structural Racism to Evaluate the Association with Preterm Birth and Infant Mortality-California, 2011-2012.* J Urban Health. , 2019. **96**: p. 159-170.
3. Agency for Healthcare Research and Quality. Creating and Validating an Index of Socioeconomic Status. http://www.ahrq.gov/qual/medicareindicators/medicareindicators3.htm (Access date: October 6.
4. Spotswood, E.N.; Benjamin, M.; Stoneburner, L.; Wheeler, M.M.; Beller, E.E.; Balk, D.; McPhearson, T.; Kuo, M.; McDonald, R.I. *Nature inequity and higher COVID-19 case rates in less-green neighbourhoods in the United States.* Nat Sustain, 2021. **4**: p. 1092–1098.
5. Trabucco, A.Z., R. Global Aridity Index (Global-Aridity) and Global Potential Evapo-Transpiration (Global-PET) Geospatial Database (CGIAR Consortium for Spatial Information, 2009.
6. Kearney DJ, K.K., Storms M, Simpson TL. , *Prevalence of Gastrointestinal Symptoms and Irritable Bowel Syndrome Among Individuals With Symptomatic Posttraumatic Stress Disorder.* J Clin Gastroenterol., 2022. **1**(7): p. 592-596.
7. Ng QX, S.A., Loke W, Venkatanarayanan N, Lim DY, Yeo WS. , *Systematic review with meta-analysis: The association between post-traumatic stress disorder and irritable bowel syndrome.* . J Gastroenterol Hepatol., 2019. **34**: p. 68-73.
8. Bugaev N, B.B., Chiu WC, Como JJ, Cripps MW, Ferrada P, Gelbard RB, Gondek S, Kasotakis G, Kim D, Mentzer C, Robinson BRH, Salcedo ES, Yeh DD. , *Antimotility agents for the treatment of acute noninfectious diarrhea in critically ill patients: A practice management guideline from the Eastern Association for the Surgery of Trauma.* J Trauma Acute Care Surg., 2019. **87**: p. 915-921.
9. Jain M, Wylie WP. Diphenoxylate and Atropine. [Updated 2023 Jun 24]. In: StatPearls [Internet]. Treasure Island (FL): StatPearls Publishing; 2023 Jan-. Available from: https://www.ncbi.nlm.nih.gov/books/NBK559300/.
10. Higdon E, T.J., Sands C. , *Moxifloxacin-induced visual hallucinations: A case report and review of the literature.* J Pharm Pract., 2016.
11. Mazhar F, A.S., Haider N. , *Moxifloxacin-induced acute psychosis: A case report with literature review.* . J Res Pharm Pract., 2016. **5**: p. 294-296.
12. Sankaran P, K.A., Tariq SM, Ruffell H, Smith AC, Prentice P, Subramanian DN, Musonda P, Myint PK. , *Are shock index and adjusted shock index useful in predicting mortality and length of stay in community-acquired pneumonia?* Eur J Intern Med., 2011. **22**: p. 282-5.
13. AM., F., *A brief review of moxifloxacin in the treatment of elderly patients with community-acquired pneumonia (CAP).* Clin Interv Aging. , 2007. **2**: p. 179-87.
14. Beutner KR, F.D., Forszpaniak C, Andersen PL, Wood MJ. , *Valaciclovir compared with acyclovir for improved therapy for herpes zoster in immunocompetent adults.* . Antimicrob Agents Chemother., 1995. **39**: p. 1546-53.
15. Yao H, Z.C., Liu L, Hu H. , *Different dosages of valaciclovir for the treatment of herpes zoster in adults: A randomized clinical study.* J Clin Pharm Ther., 2021. **46**: p. 717-723.

16. Singh R, Emmady PD. Rivaroxaban. [Updated 2023 Apr 17]. In: StatPearls [Internet]. Treasure Island (FL): StatPearls Publishing; 2023 Jan-. Available from: https://www.ncbi.nlm.nih.gov/books/NBK557502/.
17. Jentzsch T, M.R., Neuhaus V, Hussein K, Farei-Campagna J, Seifert B, Simmen HP, Werner CML, Osterhoff G. , *Is rivaroxaban associated with higher morbidity and mortality in patients with traumatic head injuries? A retrospective cohort study comparing rivaroxaban, no anticoagulation, and phenprocoumon.* Clin Neurol Neurosurg., 2018(169): p. 116-120.
18. Gupta MA, J.P., Gupta AK. , *Posttraumatic stress disorder (PTSD) and the dermatology patient.* Clin Dermatol, 2017. **35**: p. 260-266.
19. Wolf K, S.S., *The use of sodium sulfacetamide in dermatology.* Cutis., 2015. **96**: p. 128-30.
20. Miller, S.A.; Mancuso, C.A.; Boutin-Foster, C.; Michelen, W.; McLean-Long, C.; Foote, B.; Charlson, M.E. *Associations between posttraumatic stress disorder and hemoglobin A1(C) in low-income minority patients with diabetes.* Gen Hosp Psychiatry., 2011. **33**: p. 116-122.
21. Jain R, G.A., Narnoli S (2020) Haematological Changes in Alcohol and Substance Use Disorders- An Overview. Int Arch Subst Abuse Rehabil 2:006. doi.org/10.23937/2690-263X/1710006, *Haematological Changes in Alcohol and Substance Use Disorders- An Overview.* Int Arch Subst Abuse Rehabil 2020. **2**.
22. Kuperman, A.; López-Reyes, R.; Lopez-Saez, J.B.; Lorenzo, A.; Bascuñana, J.; Farge Bancel, D.; Alfonso, M.; Lumbierres, M.; Stemer, G.; Monreal Bosch, M.; Braester, A.; RIETE Investigators. *Anemia and bleeding in patients receiving anticoagulant therapy for venous thromboembolism.* J Thromb Thrombolysis., 2018. **45**(3): p. 360-368.
23. Akaoka Y, Y.H., Kodaira H, Kato H. , *Risk factors for the effect of anticoagulant and antiplatelet agents on perioperative blood loss following proximal femoral fractures. .* Medicine (Baltimore). 2016. **95**(27).
24. Lindqvist, D.; Mellon, S.H.; Dhabhar, F.S.; Yehuda, R.; Grenon, S.M.; Flory, J.D.; Bierer, L.M.; Abu-Amara, D.; Coy, M.; Makotkine, I.; Reus, V.I.; Aschbacher, K.; Bersani, F.S.; Marmar, C.R.; Wolkowitz, O.M. *Increased circulating blood cell counts in combat-related PTSD: Associations with inflammation and PTSD severity. .* Psychiatry Res., 2017. **258**: p. 330-336.
25. Chen, S.; Xia, H.S.; Zhu, F.; Yin, G.Z.; Qian, Z.K.; Jiang, C.X.; Gu, X.C.; Yin, X.Y.; Tang, W.J.; Zhang, T.H.; Wang, J.J.; Jia, Q.F.; Hui, L. Association between decreased serum albumin levels and depressive symptoms in patients with schizophrenia in a Chinese Han population: A pilot study. Psychiatry Res. 2018;270:438-442. doi:10.1016/j.psychres.2018.10.012, *Association between decreased serum albumin levels and depressive symptoms in patients with schizophrenia in a Chinese Han population: A pilot study.* Psychiatry Res., 2018. **270**: p. 438-442.
26. Kotoh, K.; Fukushima, M.; Horikawa, Y.; Yamashita, S.; Kohjima, M.; Nakamuta, M.; Enoji, M. *Serum albumin is present at higher levels in alcoholic liver cirrhosis as compared to HCV-related cirrhosis.* Exp Ther Med. , 2012. **3**: p. 72-75.
27. Lyons O, W.B., Bennett K, O'Riordan D, Silke B. , *Serum albumin as an outcome predictor in hospital emergency medical admissions. .* Eur J Intern Med. , 2010. **21**(1): p. 17-20.
28. Mao Y, L.X., Zhu S, Ma J, Geng Y, Zhao Y. , *Associations between urea nitrogen and risk of depression among subjects with and without type 2 diabetes: A nationwide population-based study.* Front Endocrinol (Lausanne), 2022(13): p. 985167.
29. Sumathi T, N.D.S., *Effect of Bacopa monniera on liver and kidney toxicity in chronic use of opioids. .* Phytomedicine, 2009(16): p. 897–903.
30. Jung S.J., K.J.H., Roberts A.L., Nishimi K., Chen Q., Sumner J.A., Kubzansky L., Koenen K.C. , *Posttraumatic stress disorder and incidence of thyroid dysfunction in women.* Psychol. Med., 2019. **49**: p. 2551–2560.
31. Meenashi Sundaram D, M.V., Rambrahma Reddy D, Baliga K., *Dyselectrolytemia in a Chronic Alcohol Abuser: A Case Report.* Cureus., 2023. **15**.
32. Sharma S., H.M.H., Aggarwal S. Hyperchloremic Acidosis. Stat pearls; Treasure Island, FL, USA: 2021.
33. Singh V., K.S., Gupta P. , *Blood gas analysis for bedside diagnosis.* Natl. J. Maxillofac. Surg., 2013. **4**: p. 136–141.
34. Yousuf T, B.T., Kramer J, Khan B, Ziffra J, Villines D, Shah P, Hanif T. , *Correlation Between Partial Pressure of Arterial Carbon Dioxide and End Tidal Carbon Dioxide in Patients with Severe Alcohol Withdrawal.* Ochsner J., 2015. **15**: p. 418-22.
35. Nowotny, B.; Cavka, M.; Herder, C.; Löffler, H.; Poschen, U.; Joksimovic, L.; Kempf, K.; Krug, A.W.; Koenig, W.; Martin, S.; Kruse, J. *Effects of acute psychological stress on glucose metabolism and subclinical inflammation in patients with post-traumatic stress disorder. .* Horm Metab Res., 2010. **42**(10): p. 746-753.
36. Ojo O, W.X., Ojo OO, Ibe J. , *The Effects of Substance Abuse on Blood Glucose Parameters in Patients with Diabetes: A Systematic Review and Meta-Analysis.* Int J Environ Res Public Health., 2018. **15**(12): p. 2691.

37. Shafiee, M.; Tayefi, M.; Hassanian, S.M.; Ghaneifar, Z.; Parizadeh, M.R.; Avan, A.; Rahmani, F.; Khorasanchi, Z.; Azarpajouh, M.R.; Safarian, H.; Moohebati, M.; Heidari-Bakavoli, A.; Esmaeili, H.; Nematy, M.; Safarian, M.; Ebrahimi, M.; Ferns, G.A.; Mokhber, N.; Ghayour-Mobarhan, M. *Depression and anxiety symptoms are associated with white blood cell count and red cell distribution width: A sex-stratified analysis in a population-based study.* Psychoneuroendocrinology. , 2017. **84**: p. 101-108.
38. Garg G, C.N., Gogia A, Kakar A. , *Low backache in adults as an initial presentation of acute lymphoblastic leukemia.* . J Family Med Prim Care. , 2017. **6**(2): p. 434-436.
39. Puangsri P, N.-A.P., *Potential usefulness of complete blood count parameters and inflammatory ratios as simple biomarkers of depression and suicide risk in drug-naive, adolescents with major depressive disorder.* . Psychiatry Res., 2021. **305**.
40. Haliloğlu S, C.A., Sahiner E, Karaaslan Y, Kosar A. , *Mean platelet volume in patients with fibromyalgia.* Z Rheumatol., 2014. **73**(8): p. 742-745.
41. Rabah H, I.A., Chalhoub M. Leukocytes in Critical Patients With Asthma Exacerbation. Cureus. 2021;13(12):e20520. Published 2021 Dec 19. doi:10.7759/cureus.20520.
42. Moalem G, T.D., *Immune and inflammatory mechanisms in neuropathic pain.* . Brain Res Rev., 2006. **51**(2): p. 240-264.
43. Rittner HL, B.A., *Leukocytes as mediators of pain and analgesia.* Curr Rheumatol Rep., 2007. **9**(6): p. 503-510.
44. K., K., *The women with dysuria.* Am Fam Physician., 1998. **57**(9): p. 2155-2170.
45. Maserejian NN, K.V., Miyasato G, McVary KT, McKinlay JB. , *Are physical activity, smoking and alcohol consumption associated with lower urinary tract symptoms in men or women? Results from a population based observational study.* . J Urol., 2012. **188**(2): p. 490-495.
46. Ismail D, M.V., Ladoyanni E. , *Focal Hyperhidrosis Associated with Recurrent Urinary Tract Infections.* . Case Rep Dermatol Med., 2016.
47. Gosling P, S.A., *Proteinuria following trauma.* Ann Clin Biochem., 1986. **23**: p. 681-685.
48. Nazrul Islam SK, J.H.K., Ahmed A, Ahsan M. , *Nutritional status of drug addicts undergoing detoxification: prevalence of malnutrition and influence of illicit drugs and lifestyle.* Br J Nutr., 2002. **88**(5): p. 507-513.
